# Diagnostic Testing for SARS-CoV-2 Infection

**DOI:** 10.1007/s11901-021-00567-9

**Published:** 2021-10-28

**Authors:** Emmanuel Thomas, Stephanie Delabat, David M. Andrews

**Affiliations:** 1 Department of Microbiology & Immunology, University of Miami Miller School of Medicine, Miami, FL USA; 2 Schiff Center for Liver Disease, University of Miami Miller School of Medicine, 1550 NW 10th Ave., Papanicolaou Bldg., RM PAP 514, Miami, FL 33136 USA; 3 Department of Pathology, University of Miami Miller School of Medicine, Miami, FL USA

**Keywords:** SARS-CoV-2, COVID-19, PCR, Antigen, Antibody, Nucleic acids

## Abstract

**Purpose of Review:**

Given the rapid development of diagnostic approaches to test for and diagnose infection with SARS-CoV-2, many options are available to assess infection. Multiple established diagnostic companies are now providing testing platforms whereas initially, testing was being performed with simple PCR-based tests using standard laboratory reagents.

**Recent Findings.:**

Additional testing platforms continue to be developed but challenges with testing, including obtaining testing reagents and other related supplies, are frequently encountered. With time, the testing supply chain will improve and more companies will be providing materials to support these testing efforts. In the USA, the need for rapid assay development and subsequent approval through attainment of emergency use authorization (EUA) has superseded the traditional arduous diagnostic testing approval workflow mandated by the FDA. It is anticipated that the USA will be able to continue to significantly increase its testing capabilities to address this pandemic; however, challenges remain due to the diversity of the performance characteristics of tests being utilized.

**Summary:**

This review provides an overview of the current diagnostic testing landscape, with pertinent information related to SARS-CoV-2 virology and antibody responses, that is available to diagnose infection.

## Introduction

Viruses can be separated into two distinct groups: those that cause persisting/chronic infection and those that only cause acute infection and are subsequently cleared by the host innate and adaptive immune responses [1]. Coronaviruses predominantly cause acute infection based on our current understanding of these viruses [2]. During acute infection, they can possibly cause mortality [3]. That immune response can provide protection against exposure to the same virus to prevent clinically significant reinfection; however, this may not be the case in some patients [4]. The degree of protection will depend on the length of time after the initial recovery since, as with non-persisting virus, immune responses wane with time [5]. In general with acute viruses, individuals may become infected again only after a long interval but usually the severity of the infection is limited [6, 7]. The degree of protection can also depend on the degree of any antigenic shift between the virus that caused the first infection when compared to that responsible for the second infection [8].

Given this background, testing for SARS-CoV-2 predominantly relies on testing for evidence of active infection through detection of viral nucleic acids or viral antigens, whereas chronic infections can most easily, at a reduced cost, be initially detected by the presence of antibodies targeting viral proteins [9, 10••]. SARS-CoV-2 testing will have to be expanded for us to adequately address the pandemic. Some estimate that the USA will require testing 3 to 4 million individuals per day to adequately address the pandemic; however, we are only testing approximately 1 million individuals per day at this time (https://coronavirus.jhu.edu/testing). This is underscored by the fact that SARS-CoV-2 has become endemic to some regions and healthcare facilities within the USA [11].

Given the rapid development of diagnostic approaches to test for SARS-CoV-2, testing will be much more robust in the future with more options available to assess infection and subsequently prevent virus spread. Multiple established diagnostic companies are now providing testing platforms including Cepheid, Genmark, Hologic, Roche, and Abbott [12], whereas initially, testing was being done with simple molecular PCR-based tests using standard laboratory reagents. In addition to challenges with obtaining adequate testing reagents, testing can also be limited by the lack of other supplies including personal protective equipment (PPE), nasal swabs, and associated testing reagents including viral transport media (VTM) [13]. With time, the testing supply chain will improve and more companies will be providing materials and products to support these testing efforts that are desperately needed. At this time, the increase in the number of new testing platforms appears to be an additive process due to the high demand to increase testing volume as opposed to a competitive process where performance dictates the use of a specific testing platform. It is important to keep in mind that the results of any test for SARS-CoV-2 will only be accurate based on their performance characteristics that can only be determined through rigorous assessment of sensitivity, specificity, positive/negative predictive value, and pre-test probabilities of active infection in a given population.

## History of COVID-19 and Other Coronaviruses that May Impact Virus Testing

**Fig. 1:**
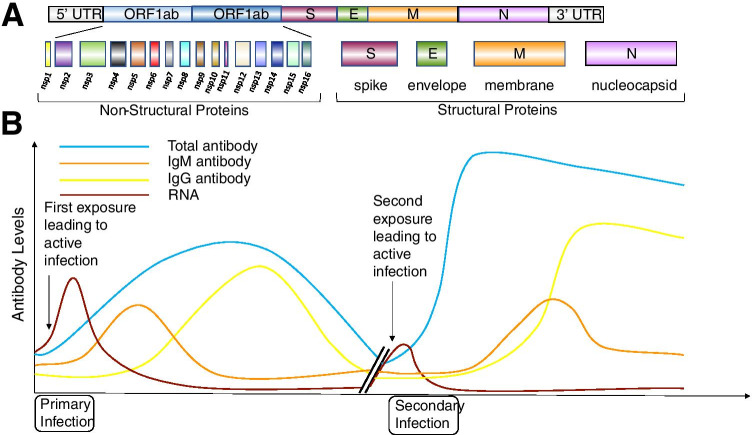
The SARS-CoV-2 genome and antibody responses. **A** SARS-CoV-2 RNA genome (30 kB) and its coding regions encoding both non-structural and structural proteins. **B** Theoretical antibody responses in humans following both primary and secondary infections with SARS-CoV-2

At this time, there have been several distinct coronaviruses discovered that infect humans and cause disease. Four of these mainly cause mild respiratory illness (229E, OC43, NL63, and HKU1) and three can cause more severe respiratory illness (SARS-CoV-1, MERS-CoV-1, and SARS-CoV-2) in a higher percentage of infected patients [14]. SARS-CoV-1 was discovered in 2003 and was the first coronavirus that frequently causes severe respiratory illness while also binding the ACE2 receptor to infect cells. However, SARS-CoV-1 was limited in its spread globally mostly to China and Hong Kong [15]. Another coronavirus was discovered in 2003 and named NL63. This coronavirus also uses the ACE2 to receptor for entry; however, this virus usually only causes mild respiratory illness but it spreads similarly to other viruses that cause the common cold [16]. The recently discovered virus, SARS-CoV-2, is an enveloped, positive-strand RNA coronavirus (Fig. 1A) that can cause severe respiratory illness and also uses the ACE2 receptor to infect cells [17]. Importantly, it also has spread globally in a similar fashion as other common cold coronaviruses but with a higher propensity to cause poor clinical outcomes [18].

Insight has been gleaned from large-scale comprehensive screening efforts into the spread of common-cold-causing coronaviruses. A study published in 2010 interrogated four common cold coronaviruses by analysis of 11,661 diagnostic respiratory samples, collected in the UK, over 3 years between July 2006 and June 2009 and they sampled patients from all age groups [19••]. It was reported that individuals usually are exposed and sero-convert from infection with these common cold coronavirus in childhood. Unfortunately, now many adults, for the first time, are being exposed to a coronavirus that is SARS-CoV-2, which is unusual when compared to exposure to the other common cold coronaviruses. Infection with common cold coronaviruses is common including 229E and OC43 that were discovered in the 1950s and 1960s and possibly cause reinfection due to waning immunity. Newer coronaviruses that also cause mild respiratory illness include NL63 and HKU1 and they likely also cause repeated infections [20]. Importantly, when scientists were developing antibody tests in 2003 for SARS-CoV-1, cross reactivity was reported between SARS-CoV-1 and samples containing either 229E or OC43 [21]. However, these non-specific test results can be rectified by additional use of multiplex assays and methods including western blot that may offer more specificity but this highlights the complexity of testing for coronavirus infection including SARS-CoV-2 [19••].

## Epidemiologic Data

The USA is currently the epicenter of the global pandemic with over 8 million cases and approximately 200,000 deaths as of October 1, 2020 (https://covid.cdc.gov/covid-data-tracker). Despite these numbers, many states in the USA are moving forward with plans to re-open businesses and schools while resuming sporting activities and it is anticipated that numbers will continue to rise for the foreseeable future (https://covid19.healthdata.org/united-states-of-america?view=infections-testing&tab=trend&test=infections) [22]. The USA is currently testing approximately 1 million individuals daily, including both asymptomatic [23] and symptomatic patients, per day for COVID-19 (https://coronavirus.jhu.edu/testing). Data suggests that the USA should endeavor to test 3–4 million individuals per day to be able to diagnose, isolate, and quarantine appropriately to mitigate the continued growth of the pandemic. Sporadic cases of reinfection with SARS-CoV-2 further complicate these testing efforts [24].

## SARS-CoV-2 Molecular Characteristics

The genome of the virus consists of a 32 kilobase RNA genome (Fig. 1A) [25]. The 5′ region encodes its non-structural proteins and its structural proteins are encoded toward the 3′ untranslated region [26]. Upon binding of SARS-CoV-2 to its receptor ACE2, it is internalized and uncoats following acidification in endocytic vesicles [27]. This acidification and uncoating is important for the virus to be able to release its genomic RNA into the cytoplasm. Once released, since the genomic RNA is positive stranded, the RNA genome can be directly translated into its viral proteins after host ribosomes bind the 5′ region and start the translation process [28].

The viral genomic RNA encodes specific structural proteins including the envelope (E), matrix (M), and the spike (S) protein. This spike protein is the specific protein through which this virus attaches to cells and enters through interactions with the ACE2 receptor. The spike glycoprotein is expressed on the outer surface of the envelope that surrounds an inner nucleocapsid that is a ribonucleoprotein. This nucleocapsid is important for interacting with the viral genome and is produced in high abundance [29].

It is important to point out that SARS-CoV-2 has multiple proteins that can generate antibody responses. A common target is the spike glycoprotein and many vaccine strategies are targeting this specific viral protein [30]. As a consequence, it is imperative that future antibody tests are generated that target antibodies to other viral proteins including the nucleocapsid protein that is produced in a large quantities by SARS-CoV-2 [31]. This will allow a test to distinguish between patients that are vaccinated against the virus spike protein and those individuals that have been exposed to SARS-CoV-2 but are unvaccinated.

Figure 1B is a diagram depicting a theoretical antibody response profile of someone that has been exposed to SARS-CoV-2 [32]. When an individual is first exposed to SARS-CoV-2, the viral RNA becomes detectable and over time, the patient subsequently generates both IgM and IgG antibodies that may control the virus and lead to a decrease in circulating viral genomes to undetectable levels [33•]. If a second infection is encountered with the same virus that has not undergone significant antigenic shift, then a more robust antibody response may be produced that should more quickly control this second infection [34]. However, we do not have rigorous evidence if this happens in all patients exposed to SARS-CoV-2 but it can be used to understand antibody testing and the possibility of reinfection [35].

Importantly, antibody testing in immunocompromised individuals can be more complex [36]. There is evidence that antibody responses are impaired in older individuals [37], in persons living with HIV, and in other immunocompromised populations [38]. Both B and T-cell compartments are adversely affected in these individuals and testing may be needed a second time several weeks after an initial test, if it is negative, to confirm that the patient is indeed antibody negative. In addition, multiple testing approaches will most probably be needed to appropriately characterize immune responses in these individuals. Determining the ability to generate lasting antibody responses and ascertaining prevalence data from the community, especially in individuals vaccinated for SARS-CoV-2, will be important in understanding the scale of the pandemic, future vaccine utility, and prospects for achieving herd immunity [39].

## COVID-19 Symptoms

At this time, testing for active COVID-19 infection (nucleic acid or antigen) is primarily being done in individuals with symptoms, those with known exposures, in healthcare settings and as surveillance in high risk environments including schools and nursing homes [40•]. Since, there is a higher risk of poor clinical outcomes in individuals that are older in age and that have serious chronic health conditions, it is important to test these patient populations when COVID-19 is suspected [41•]. Signs and symptoms of COVID-19 include those associated with other respiratory viruses including influenza; however, some symptoms affect other organ systems in COVID-19. Typical symptoms of respiratory virus infection include fever/chills, cough, shortness of breath, difficulty breathing, fatigue, muscle/body aches, headache, sore throat, and congestion or runny nose. Moreover, COVID-19 specific symptoms may include new onset of loss of taste/smell, nausea/vomiting, and diarrhea as well as multisystem inflammatory syndrome in children (MIS-C) with COVID-19 [42]. Given that many of these symptoms can overlap with influenza and respiratory syncytial virus (RSV) infection, it is important to also test for these viruses especially during flu season [40•, 43].

## Time Range of Infectious Period and Clearance

At this time, 10 to 14 days is the standard for an appropriate quarantine period for COVID-19 to ensure minimal spread of the virus based on viral load measurements and symptomatic presentation [44]. Therefore, the overall testing window can be 2 to 12 days following an exposure. Optimally, testing can be considered 5 to 7 days following an exposure with 7 days post exposure being favored. Patients usually present with symptoms 2 to 5 days following an exposure [45] and can be virus positive 1 to 2 days before symptom onset; therefore, it is better to conduct testing as soon as symptoms arise, or as close to a known exposure as possible, so not to progress too far from the day of exposure (https://www.cdc.gov/coronavirus/2019-ncov/symptoms-testing/symptoms.html). If an individual has had symptoms and has recovered, it may be more appropriate to test for antibodies to determine if an individual was indeed exposed to SARS-CoV-2 as opposed to testing for active infection through a nucleic acid or antigen test [40•].

## Diagnostic Testing Overview

The US Food and Drug Administration (FDA) heavily regulates diagnostic testing to diagnose viral infections. For device and test kit manufacturers, obtaining FDA diagnostic testing approval usually involves a long process of validation and comparison studies. Similarly, clinical laboratories that seek FDA approval must meet rigorous standards to obtain FDA approval. Due to the public health need with the current pandemic, many SARS-CoV-2 diagnostic tests have been approved by the FDA for emergency use authorization (EUA) after limited validation studies have been conducted [46]. This has led to a wide range of performance characteristics between tests. Notably the FDA EUA status of a particular test is considered temporary, only being valid during the time period associated with the national health emergency. After the emergency, the FDA reserves the right to revoke FDA EUA approval, requiring the manufacturer to perform additional studies to obtain full FDA approval.

In addition to manufacturers, during the first several months of the COVD-19 pandemic, clinical reference laboratories and hospital-based laboratories submitted applications for FDA EUA approval for SARS-CoV-2 tests. In order to decompress the overwhelming demand for EUA test reviews, the FDA permitted high-complexity CLIA-certified laboratories to perform validations of their internally developed SARS-CoV-2 tests as laboratory developed tests (LDTs). Overall quality and performance characteristics are defined by the laboratory accrediting agency, such as the College of American Pathologists (CAP).

There can also be problems with testing not only based on the characteristics of the test, but also with the sample that is obtained. Considerations for sample procurement include ensuring that the sample is appropriate and adequate to contain sufficient viral material to be detected by the assay being employed [47]. Also the sensitivity and specificity of the tests must be considered for the tests that are being used; however, due to obtaining EUA approval, adequate information may not be available when compared to FDA-approved tests used to diagnose other viruses [48•]. With time, only testing using the most rigorous approaches in the future will likely continued. At this time, the medical community is dependent on testing from facilities that have appropriate infrastructure to conduct adequate testing; however, it is difficult to ensure the rigor and reproducibility that is available in all testing laboratories, with the plethora of tests for SARS-CoV-2, that is observed with testing for other viruses [10••]. In addition, the lack of availability of standardized testing reagents with FDA approval also contributes to these challenges with testing [46, 48•].

## General Virus Testing Approaches

There are several approaches used to test for SARS-CoV-2 [49]. Molecular testing involves the detection of viral nucleic acid, after amplification, and test results can determine whether or not a patient has active infection that may be transmissible depending on the viral load. Most technologies utilize polymerase chain reaction (PCR) that requires temperature variation and can take longer from sample input to result. PCR is a frequently used molecular method for virus detection due to its high sensitivity. Importantly, PCR-based tests are also more amenable to provide quantitative results pertaining to viral load [50]. Newer, non-PCR-based methods utilize approaches that can facilitate rapid identification of nucleic acids using technologies leveraging isothermal amplification [51, 52]. These tests are more amenable to use in the point-of-care setting given that they have the ability to provide a rapid, qualitative result. Molecular testing is more costly due to the need to utilize delicate nucleic acid polymerases that are heat-labile enzymes that drive the amplification reactions needed for sample detection [53]. At this time, there are no genotyping tests needed due to lack of variation in SARS-CoV-2 that is encountered with other viruses including HIV and HCV and it does not appear that antivirals select for resistant mutants either at this time [54, 55].

An additional approach utilized to diagnose active infection involves technologies that are capable of detecting viral antigens [56, 57]. These tests can rapidly detect various viral proteins including the SARS-CoV-2 spike and nucleocapsid and the results can be read and documented by smartphones to report test results to interested parties. Viral antigen testing is standard practice for influenza and RSV testing and is usually done with samples obtained from a nasopharyngeal swab but it can also be performed on blood samples [56, 58]. These antigen tests tend to also be less expensive than molecular assays given the lower cost of the associated reagents for this testing platform. However, the sensitivity of this technique remains low compared to other methods for SARS-CoV-2 testing. Interestingly, this lower sensitivity may be useful in screening efforts, especially in asymptomatic individuals, since a “low” positive PCR test results may indicate infection in an individual unlikely to be able to spread the virus to others.

In addition to approaches to diagnose active virus infection, there are also serologic testing approaches [31]. Typical serologic tests focus on the detection of human antibodies recognizing viral proteins and these include IgM, IgG (Fig. 1B), or total antibody levels [59•]. These antibody tests provide insight as to whether or not an individual has been exposed to a virus [60]. These tests can be lower in cost and they can also be amenable to rapid point-of-care testing from either blood or saliva. However, generation of these tests take longer because they require biologic reagents including viral protein antigens and also capture antibodies. This differs from nucleic acid testing where PCR primers can be generated very quickly that are very sensitive and specific for a distinct viral genome. Table 1 describes the utilization of these tests in the clinical evaluation of a patient that is suspected to be infected with SARS-CoV-2.

**Table 1:** Testing workflow for the SARS-CoV-2 virus in patients suspected of having active infection

SARS-CoV-2 testing algorithm					
#1 Signs and symptoms		#2 Diagnostic testing		#3 Follow-up	
Primary	Additional	Qualitative	Quantitative	Positive test result	Negative test result
Recent onset of acute respiratory symptoms including: • Sore throat • Cough • Shortness of breath	• Fever • Diarrhea • Vomiting • Recent loss of smell or taste • Chills • Muscle fatigue	• Isothermal nucleic acid amplification assay • Antigen detection assay • Viral sequencing assay	One-step or multiple-step RT-PCR assay	Subsequent monitoring • Report positive findings following reporting guidelines • Emphasize prevention measures to limit spread (isolation/quarantine) • Consider therapeutic intervention for more severe symptoms	Consider possible false negative result • Exposure history • Other clinical findings • Antigen test was performed • Perform antibody test if available to document possible exposure

## Testing Site

It is important to note that for testing for SARS-CoV-2, that site of sample acquisition is a large determinant of test performance. For nucleic acid testing, sensitivity can vary greatly. In symptomatic patients, nasopharyngeal swabs are more sensitive (63%) than oropharyngeal swabs (32%) while bronchoalveolar lavage fluid specimens are the most sensitive (93%). It appears that SARS-CoV-2 may move from the upper airway to the lower airway with disease progression and the presence of more severe symptoms. Testing samples from multiple sites may improve sensitivity and reduce false negative results. Risks of false negatives and testing turnaround time are important considerations. Testing patients with clear symptoms of COVID-19 infection can improve test performance with adequate sample acquisition. Saliva testing is being used more routinely since sample processing strategies have been developed that improve nucleic acid release from virions while also utilizing proteinase K to process more viscous samples [61•].

## Molecular Testing (Multi-step vs. One-step and Quantitative vs. Qualitative)

For molecular testing, there are a variety of platforms currently being used [62••]. The simplest nucleic acid tests involve only a few steps including sample acquisition straight to sample analysis and subsequent test result [61•]. These tests can be quantitative as is the molecular PCR-based test run on the Cepheid GeneXpert (45 min-PCR) or qualitative as from the Abbott ID Now (15 min-isothermal). PCR tests can take longer to perform given the need for multiple cycles at different temperatures but the result can yield semi-quantitative results by providing a cycle threshold (CT) that can be useful clinically in making decisions to prevent nosocomial spread in healthcare settings [63].

In addition to these simple tests, other EUA approved diagnostic assays may require additional separate processing steps that include viral RNA isolation and cDNA synthesis in addition to the standard amplification and detection steps [62••]. The addition of sample processing steps increases the time needed to complete the test and can also be a source or variation in test performance. In addition, these multiple steps can be automated or done manually adding additional variation in test performance [62••]. A newer EUA approved platform is among the most sensitive and uses droplet digital (dd)PCR that can detect 5 units/copies of SARS-CoV-2 in 1 ml for a partitioned sample [64]. However, to achieve a lower limit of detection, this platform requires multiple sample processing steps and is more expensive to perform when compared to standard PCR-based testing [12].

## Antigen Tests

More recently, SARS-CoV-2 viral antigen tests have received EUA approval and this is a significant addition to the testing capabilities available in the USA [57]. Antigen testing has been used for many other viruses including influenza and RSV from nasopharyngeal samples and denotes active infection [56, 58]. Since there is no amplification process, antigen testing is amenable to rapid point-of-care settings. Although the sensitivity of these tests may not be as high as seen with molecular test, they usually are lower in cost to perform. In addition, given that the sensitivity is not as high, they are amenable for use in screening programs since individuals that are antigen positive may have corresponding higher viral loads at the time of testing and may also be more infectious requiring isolation and/or quarantine [65]. For negative testing results on symptomatic patients in healthcare settings, a reflex molecular test should be performed due to the lower sensitivity of antigen tests (Table 1). There are several antigen testing platforms available from established companies including Becton Dickinson, Abbott, and Quidel and they are straightforward sample-in result-out platforms.

## Serology/Antibody Tests

Antibody tests are frequently used in the diagnosis of viral infections especially as a lower-cost option to screen for chronic viral infections [66]. The potential utility of antibody tests are numerous [31]. They can facilitate the detection of SARS-CoV-2 in recently infected patients who present late in their disease course with very low viral loads beneath the detection limit of molecular assays [67]. This is also important when lower respiratory tract sampling is not possible because upper airway secretions, including saliva, may not contain as much viral RNA as seen in the lower respiratory tract of infected individuals as the disease progresses (Table 1). By identifying the presence of these antibodies, the identification of potential convalescent plasma donors can also be accomplished [68]. It will also allow verification of functional vaccine responses once antibody correlates of protection are indeed verified. Antibody testing may also support the identification of healthcare workers that can have some protection from future infection through the presence of neutralizing antibodies since it is very important to limit nosocomial spread of SARS-CoV-2 [31, 60, 63]. Importantly, as we think about chronic illnesses in the setting of COVID-19 disease, these antibody tests can support the identification of patients that may have future disease exacerbations of chronic organ illnesses following an exposure and resolution to SARS-CoV-2 infection particularly when chronic lung disease is present before infection [69].

There are also potential drawbacks to these serologic assays if they are not well validated before use [59•]. False negatives may be found if performed early in the disease course as IgM may only develop as early as 1 week after exposure. False negatives in this case result from patients that were tested too early before they have developed detectable antibody responses. They may also occur in patients that only developed very mild disease that did not progress systemically and was limited only to the upper airway. False positives are also a risk particularly with tests for IgM due to potential cross-reactivity with common cold coronaviruses that were already mentioned [19••]. Also if SARS-CoV-2 spike protein is going to be used in many vaccines, this will necessitate testing for other viral antigens including the nucleocapsid protein in the future to distinguish between vaccinated and naturally exposed patients as previously mentioned.

Antibody testing platforms that are currently being used include lateral flow assays that utilize chromatographic strips for testing [70]. This is commonly used in tests from companies including Orasure that have tests available for both HIV and HCV antibody testing. These CLIA waived tests are performed individually as point-of-care tests and they can give results in less than 20 min. These tests can be performed in the community or as home-based tests, but they tend to be higher in costs of approximately $20 dollars each using this methodology [71, 72]. An additional platform uses the ELISA technology that is more amenable to high-throughput screening [49, 67]. Screening using the ELISA platform can be even lower in cost at approximately $5 per test. Both of these tests just require serum or plasma but ELISA can be done in an automated high-throughput format. There are automated instruments in many CLIA-certified laboratories available to be used for antibody testing and these solid-phase tests can be performed even in a higher-throughput, low-cost workflow.

Overall, the antibody response targeting SARS-CoV-2 in infected patients remains largely uncharacterized for breadth and potency and research is currently underway to clarify this issue [32, 34]. Differences in the generation of neutralizing antibodies against SARS-CoV-2 between individuals have been observed and these differences are likely important. False positives and negatives remain a concern in commercially available tests with EUA designation by the FDA in the USA [67]. Importantly, serology tests are now being developed from established companies and these tests will likely have higher sensitivity and specificity than current tests that may be available from newer companies in this space.

## Conclusion

Given the rapid emergence of the COVID-19 pandemic, the global diagnostic community has rapidly and efficiently developed testing strategies to detect many components of the SARS-CoV-2 virion. When compared to other pandemics, the global efforts to develop and improve testing capabilities for this deadly virus are unparalleled [10••]. However, in the USA, the need for rapid assay development and subsequent approval through attainment of EUA has superseded the traditional long and arduous diagnostic testing approval workflow mandated by the FDA. It is anticipated that technology development to facilitate testing for COVID-19 will positively impact diagnostic capabilities for other viruses. For example, there is still no FDA-approved point-of-care nucleic acid or antigen test for hepatitis C, which is the most common chronic bloodborne infection in the USA In less than 1 year, these tests have been developed for COVID-19 and have received EUA approval. Clearly, these efforts were supported by the availability of increased resources from multiple parties including governments, the private sector, and diagnostic companies that include those that were not traditionally involved in virus testing. All of these efforts are contributing to the steady increase in the ability to test for SARS-CoV-2. It is anticipated that the USA will be able to significantly increase the testing capability to 100 million per month that will support efforts to keep the economy open while limiting the spread and subsequent poor clinical outcomes associated with COVID-19.
